# Curation of viral genomes: challenges, applications and the way forward

**DOI:** 10.1186/1471-2105-7-S5-S12

**Published:** 2006-12-18

**Authors:** Urmila Kulkarni-Kale, Shriram G Bhosle, G Sunitha Manjari, Manali Joshi, Sandeep Bansode, Ashok S Kolaskar

**Affiliations:** 1 Bioinformatics Centre, University of Pune, Pune 411 007 India

## Abstract

**Background:**

Whole genome sequence data is a step towards generating the 'parts list' of life to understand the underlying principles of Biocomplexity. Genome sequencing initiatives of human and model organisms are targeted efforts towards understanding principles of evolution with an application envisaged to improve human health. These efforts culminated in the development of dedicated resources. Whereas a large number of viral genomes have been sequenced by groups or individuals with an interest to study antigenic variation amongst strains and species. These independent efforts enabled viruses to attain the status of 'best-represented taxa' with the highest number of genomes. However, due to lack of concerted efforts, viral genomic sequences merely remained as entries in the public repositories until recently.

**Results:**

VirGen is a curated resource of viral genomes and their analyses. Since its first release, it has grown both in terms of coverage of viral families and development of new modules for annotation and analysis. The current release (2.0) includes data for twenty-five families with broad host range as against eight in the first release. The taxonomic description of viruses in VirGen is in accordance with the ICTV nomenclature. A well-characterised strain is identified as a 'representative entry' for every viral species. This non-redundant dataset is used for subsequent annotation and analyses using sequenced-based Bioinformatics approaches. VirGen archives precomputed data on genome and proteome comparisons. A new data module that provides structures of viral proteins available in PDB has been incorporated recently. One of the unique features of VirGen is predicted conformational and sequential epitopes of known antigenic proteins using in-house developed algorithms, a step towards reverse vaccinology.

**Conclusion:**

Structured organization of genomic data facilitates use of data mining tools, which provides opportunities for knowledge discovery. One of the approaches to achieve this goal is to carry out functional annotations using comparative genomics. VirGen, a comprehensive viral genome resource that serves as an annotation and analysis pipeline has been developed for the curation of public domain viral genome data . Various steps in the curation and annotation of the genomic data and applications of the value-added derived data are substantiated with case studies.

## Background

The emergence of high throughput technologies for genome sequencing, microarrays and proteomics transformed biology into a data-rich information science. Sequencing the complete genome of an organism is the first step in generating the 'parts list' of life. One of the first efforts involved sequencing of *Haemophilus influenzae *in 1995 [1]. As of July 2006, more than 403 organisms have been sequenced completely. Furthermore, the genome sequencing projects of ~932 prokaryotic and ~608 eukaryotic species have been launched [2]. Enormous data generated by the genome sequencing projects is archived in both dedicated genomic resources and public domain databases. While the complete genome sequencing of the model organisms and microbes are taking the center-stage, viral genome sequencing continue to be individual efforts [3]. Viruses are a diverse group of organisms and are most abundant [4,5]. The genome size of viruses varies from a few hundreds to millions of bases [6,7]. *SV-40 *was the first virus for which the complete genome (5,224 bp) sequence was obtained in late 70s [8]. About ~4000 viruses have been sequenced so far by virologists all over the world with an objective to study antigenic variation, geographic distribution, spread and evolution. These independent efforts enabled viruses to attain the status of 'best-represented taxa' with the highest number of whole genomes sequenced. However, due to lack of concerted efforts, viral genomic sequences only added to the entries in the public repositories until recently. The GOLD (Genome OnLine Database) is a tracking system for genome sequencing and provides the update of various genome-sequencing projects [2] but does not have any mechanism to specifically monitor viral genome sequencing initiatives.

Whole genome sequence data of viruses offer unlimited opportunities for data mining and knowledge discovery [9]. The complete genome sequences of two large viral genomes viz., *Mimivirus *[7] and *Polydnavirus *[10] substantiate this fact. Varying coding density and the occurrence of genes associated with metabolic pathways in these DNA viruses offers interesting opportunities in viral genomics in general and in understanding evolution of viruses in particular [11]. However, it is known that in the absence of curation and functional annotation of the genomic data, the utility of the sequence data is minimal and the sequence merely remains as an entry in the database. Bioinformatics provides large number of databases, tools and approaches for mining huge sequence data. Although there exist numerous genome databases for the model organisms and microbes, there are a few databases, which archive viral genomic data [12,13]. Most of these databases are synthesis of experimental work carried out in the respective laboratories. As a result, these compilations are highly specialized [14-18].

## Results & Discussion

### VirGen: genome annotation & comparative genomics pipeline

VirGen is developed and maintained at the Bioinformatics Centre, University of Pune and is available on-line [19,20]. VirGen has grown since its initial release wherein data pertaining to eight viral families was archived. With every release new data in terms of viral families (Table 1) and utilities for analysis have been incorporated. In addition to the guided tour, release notes and statistics, VirGen provides a sitemap, which can also be used as a starting point for navigation. A new module that archives viral protein patterns is also due for release.

The major focus of this paper is towards sharing with the research community the issues involved in curation of viral genomic data apart from demonstrating the utility of the derived data in understanding the biology, a pre-requisite for development of viral diagnostics, vaccines and drugs.

#### Current status

The release (v2.0, dated August 15, 2006) contains genomic data of 25 viral families (Table 2) that include 2895 genomes and 23121 annotated proteins. As can be seen, genomic data for both, DNA and RNA viruses infecting animals, plants and microbial species are included in VirGen.

#### Salient features

▪ Curation of genomic entries of viruses

          ◦ Organization of genomic data in a structured fashion to facilitate navigation from family to strain/isolate

          ◦ Compilation of representative genomic entries for every viral species

▪ Annotation of genomic entries

▪ Compilation of synonyms for viral proteins

▪ Graphical representation of genome organization using SVG technology

▪ Precomputed Multiple Sequence Alignments (MSA) of genomes and proteomes

▪ Reconstruction of phylogeny using genome/proteome data

▪ Prediction of sequential and conformational B-cell epitopes

▪ Curation and compilation 3D structures of viral proteins available in PDB

**Figure 1:**
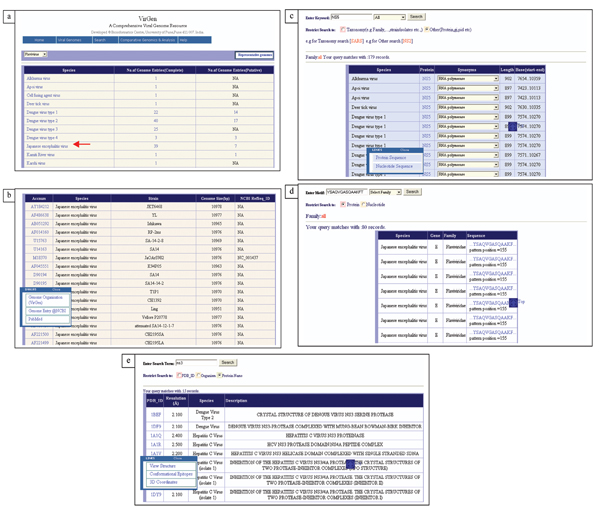
**Home page of VirGen: a genome annotation and comparative genomics platform for viruses**. (a) Snap shot of navigation via taxonomic hierarchy illustrated using complete and putative genomes of Flavivirus members. (b) Listing of various strains of Japanese encephalitis virus with complete genome. (c) A session showing result of keyword-based search (d) A session showing result of motif-based search (e) An interface to search viral structures.

Figure 1 illustrates various modes by which one can navigate and retrieve data from VirGen.

### Issues involved in curation of viral genomes

Genome sequences deposited in the public domain sequence repositories were retrieved using well-defined search strategies. The queries were formatted using keywords and MeSH terms to ensure that none of the complete genome sequences were missed. The entries were curated with respect to taxonomic hierarchy as per the guidelines provided by the ICTV [21]. For many entries, the names of viral strains/isolates were not explicitly present in the field 'organism source' but were available as a part of feature table annotations. It was also observed that in case of a few entries, although the published reference explicitly documented the strain information along with the accession numbers, the same was missing in the sequence record. Such entries were curated manually.

In case of *Hepatitis C Virus *(HCV), the curation of genomic records for assignment of genotypes called for exhaustive sequence analyses. *Hepatitis C virus *is a member of family *Flaviviridae*, genus *Hepacivirus *and is a major causative agent of liver diseases. The assignment of *Hepatitis C virus *genotypes is essential as molecular epidemiology differs from subtype to subtype. Also, due to the absence of significant cross-protection among different HCV subtypes, the exact subtype identification becomes a prerequisite in determining the suitability of present antiviral therapy as well as designing new antiviral compounds and vaccines [22]. Isolation of HCV by standard immunological and virological techniques [23] is rather difficult. Efforts to classify HCV at type and subtype level are based on molecular phylogeny using 5'UTR and core, NS3, NS4 & NS5 respectively [24]. However, absence of significant sequence variation in 5'UTR limits its usage for classification only up to genotype level [25]. Furthermore, intratypic crossing over events have been reported in some of the HCV genotypes [26]. Given this scenario, use of a single gene or genomic region may lead to inaccuracies in genotype assignment of HCV. During the process of curation of HCV genomic sequences for incorporation into VirGen, it was found that a majority of the genomic records in the public domain repositories lacked the information on genotype. An approach based on whole genome phylogeny was used to assign the genotypes. These assignments were further substantiated through literature search. More than 130 records of HCV were annotated and added to VirGen using this process.

#### Identification of putative genomic records

It was observed that there were many sequences in the public domain repositories, which were not explicitly annotated with keywords such as full or whole or complete genome even though their sequence lengths were in the typical range of the complete genome sequence for a given species. Such sequences, which are not explicitly annotated as the complete genome entries have been referred to as 'putative genomes' in VirGen [19].

#### Identification of reference dataset

As there exist multiple genomic entries for every viral species, a well-annotated and characterized entry has been identified as the 'representative genomic entry' for a given species. The representative entries provide a non-redundant set of viral genome sequences, which are subsequently used for annotation of genomic records, alignment of genomes and proteomes and to study phylogenetic relationships.

#### Curation and annotation of complete genomes

VirGen uses sequence-based Bioinformatics approaches and stringent cutoffs for annotating the viral genomes and proteomes. Entries are annotated with respect to the genome organisation, typical to a given family. The annotations are further refined to accommodate genus and species-specific organisation. Using the representative genomes and the program BLAST [27], the entries are annotated with respect to individual coding sequences (cds), polyprotein(s) and individual proteins as described previously [19].

#### Compilation of synonyms for viral proteins

**Figure 2:**
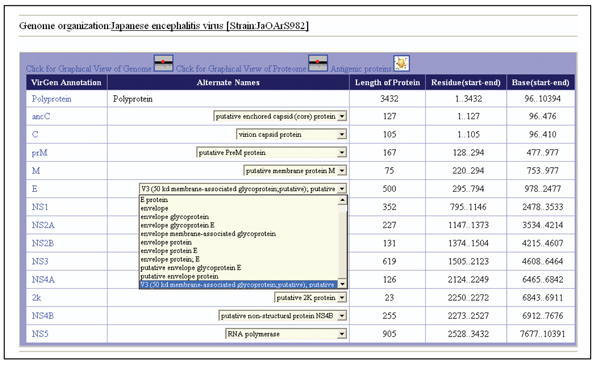
Tabular display of genome organisation of Japanese encephalitis virus depicting 'alternate names' for membrane protein.

One of the issues in curation of molecular data is lack of standard nomenclature. VirGen addresses this issue by implementing sequence-based searching to compile a dictionary of synonyms for viral proteins [19,28]. Such a compilation is essential not only in automating the annotation procedure but also to enhance quality of annotations. This dictionary of controlled vocabulary of protein names is used in the back-end for enhancing the utility of keyword-based searches in VirGen. Figure 2 shows tabular display of genome organisation along with list of alternate names for membrane protein of *Japanese encephalitis virus strain JaOArS982*.

### Derived data & its applications

#### Graphical genome view

**Figure 3:**
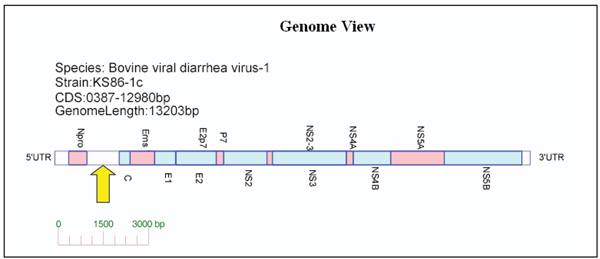
**Graphical display of genome organization of Bovine Viral Diarrhoea Virus – 1**. Insert from *Bos taurus *is shown using yellow arrow.

The schematic representation of the viral genome/proteome organisation using scalable vector graphics (SVG) has enabled visualization of individual gene/protein in the context of the entire genome. This display also facilitates retrieval of gene/protein sequence data in FASTA format, a procedure which may otherwise require a lot of pre-processing. The genome organisation of the *Bovine Viral Diahorrea Virus 1 *(BVDV-1) virus is shown in the Figure 3. As can be seen, a stretch of 996 nucleotides (891–1787 bp) do not have any annotation. A search of this sequence using BLAST against the viral division of GenBank did not provide any hit indicating a non-viral origin for the sequence. Subsequent search against the nr database picked up a few entries of J-domain containing protein from *Bos taurus*, with statistically significant scores, indicating that the sequence was acquired by BVDV-1 from its host, the bovine, through a process of horizontal transfer. BVDV is known to acquire such heterologous and homologous inserts [29].

#### Genome scale multiple sequence alignment

Comparative genomics inadvertently includes multiple sequence alignment (MSA) in a major way, as the inferences drawn are very informative. Viruses being one of the smallest replicating species are under severe selection pressure not only to evade host immune response but also to sustain its survival in the vector. Availability of genome data of viruses offers opportunities to study variations at molecular level.

Bioinformatics tools like MSA can be used to identify such variations, which when mapped on phenotypic properties like antigenicity, immunogenicity etc. may provide a rationale for the observed strain and species-specificity. MSA data and predicted epitopes for HN protein along with the predicted 3D structures were used to study strain specificity of mumps virus [30].

MSA module in VirGen is computed using the parallel version of ClustalW [31]. Multiple sequence alignment of viral genome and proteome at different levels of taxonomic hierarchy, apart from being a prerequisite for phylogenetic analysis and mapping of predicted B and T-cell epitopes, help to detect species and strain-specific signature sequences and in primer design. The MSA module can also be browsed independently to access alignments and dendrograms.

**Figure 4:**
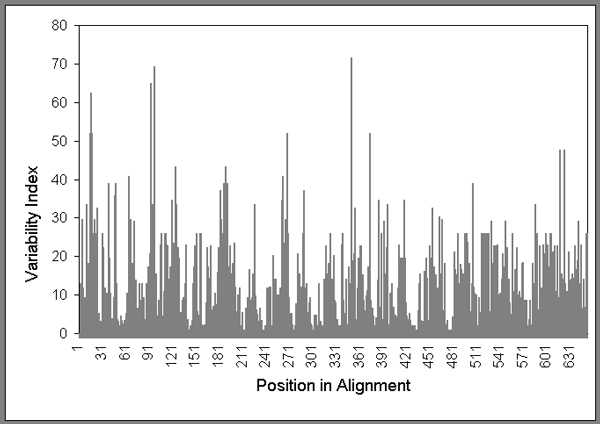
Variability Index of NS3 protein calculated for 26 species belonging to genus Flavivirus.

**Figure 5:**
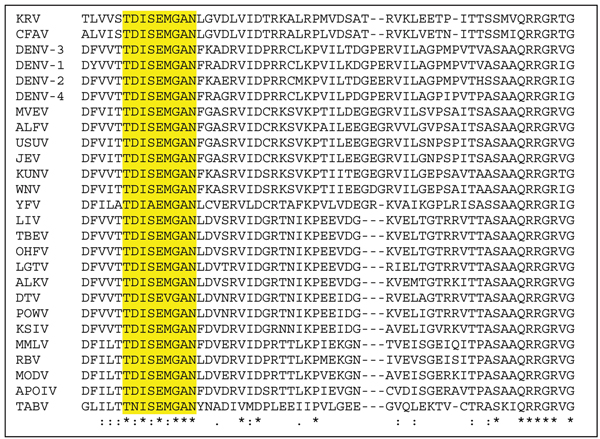
**A block of multiple sequence alignment of NS3 of 26 species belonging to genus Flavivirus**. Abbreviations of viral species are as per ICTV definitions. The genus specific pattern T- [DN]-I- [AS]-E- [VM]-G-A-N of NS3 has been derived using this MSA.

As an example, multiple sequence alignment of NS3 protein for the 26 representative species of genus *Flavivirus *is discussed. Variability Index, calculated using the formulae proposed by Wu & Kabat [32] for MSA of NS3 is shown in the Figure 4. Variability Index provides information about the extent of sequence conservation/variation at a given position in the multiple sequence alignment. As can be seen from Figure 4, the variability index values range from 1 to 71.5, wherein the value 1 indicates that the residue is conserved at a given position. A total of 64 amino acid residues spread across the alignment spanning 652 positions are conserved. The serine protease catalytic triad of NS3 comprising Histidine, Aspartate and Serine (Alignment positions 56, 80 and 141 respectively) are also conserved. The alignment position accounting for the highest variability is 349. A closer look at the alignment shows that 11 distinct amino acids viz., His, Asn, Ala, Glu, Gln, Ile, Val, Met, Thr, Ser, Cys are found to occur at that position. One of the blocks of MSA showing conserved residues is shown in Figure 5. This data was used to derive genus-specific signature 'T- [DN]-I- [AS]-E- [VM]-G-A-N' of NS3. To assess the accuracy of this pattern, sensitivity and specificity values were calculated. A search against NCBI Taxonomy revealed that there exist 84 species in genus Flavivirus, of which the amino acid sequence data for NS3 is available only for 30 species. The pattern derived using VirGen representative data, picks up all the 30 species. No false positives were picked up. Hence, the sensitivity and specificity of the pattern is 100%. The search against pattern database, Prosite [33] revealed that the reported pattern is novel.

It must be mentioned that these calculations require a curated non-redundant data set that includes NS3 protein sequences of all the known species belonging to genus *Flavivirus*. However, due to availability of limited annotations and NS3 sequence being part of polyprotein entries in the public domain databases, the curation process could not be automated completely and required manual intervention. Thus, the entire procedure of generating a curated dataset for calculation of sensitivity and specificity of any pattern is time-consuming and calls for inclusion of steps that are specific for a given data set.

**Figure 6:**
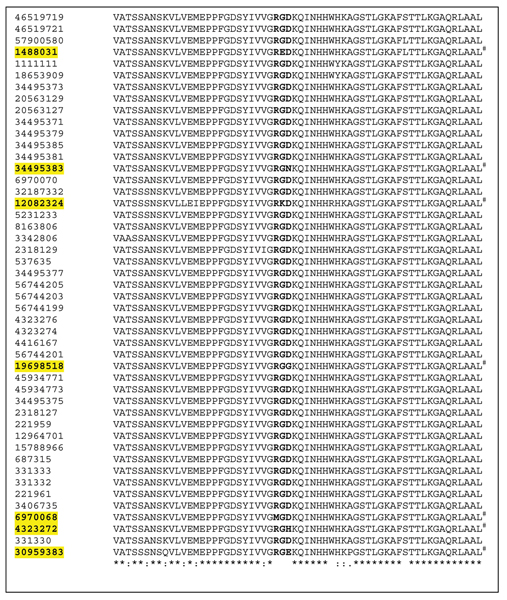
**A block of multiple sequence alignment of envelope glycoprotein of 47 strains of Japanese encephalitis virus**. Tripeptide motif RGD is shown in bold. GenPept GI numbers of the sequences with mutations in RGD are highlighted in yellow.

Multiple sequence alignment can also be used to understand the strain-specific variations. A block of multiple sequence alignment of envelope glycoprotein (Egp) of 47 strains of *Japanese encephalitis virus *(JEV) is shown in Figure 6. Important biological activities like hemagglutination, viral neutralization, virion assembly; membrane fusion and viral binding to cellular receptors are known to be associated with the 53 kD Egp protein of JEV [34-36]. Egp contains ~500 residues and folds into three structural domains [37,38]. Of the 500 residues, 318 (63.6%) are conserved, which amounts to 36.4% of variation. This variation can be attributed to high selection pressure on Egp due to its antigenicity as well as the need to maintain the strain-specific properties. Of the 182 variable positions, singletons account for 100 positions, leaving only 82 sites with information content for phylogenetic analyses. However, singleton sites like K_279_M play a deterministic role in maintaining neurovirulence [39].

Furthermore, the 399-RGD-401 sequence motif, present in domain III is unique to the mosquito-borne Flaviviruses and was proposed to form a part of receptor binding site [40,41]. The mutations found within the loop containing the RGD motif are known to alter tropism or virulence in different Flaviviruses [42]. MSA of 47 strains of JEV Egp show mutations in this region, leading to occurrence of seven unique tripeptides (RGE, RGH, RGG, RKD, RED, RGN, MGD). Curation and MSA of Egp sequences from additional 88 JEV strains revealed that IGD also occurs in place of RGD. All tripeptides except MGD and RGN are naturally occurring. MGD was observed in the attenuated strain (GenPept Accession: 6970068) [42]. Similarly RGN was observed in a strain, which was passaged in Neuro-2a cells (GenPept Accession: 34495383). The RED tripeptide is present in a highly neurovirulent and neuroinvasive strain P3 (GenPept Accession: 1488031) [43]. Thus, every variation is significant and can be correlated with functionality in the context of selection pressure.

#### Reconstruction of phylogeny using complete genomes

Molecular phylogenetic studies help to decipher the evolution of viruses and offer a mechanism to understand the origin and spread of infectious viral diseases. Phylogenetic trees are usually reconstructed using a single gene/protein or a region encompassing them [44]. UTRs have also been used to study phylogenetic relationships [45]. It was shown that the phylogenetic noise is minimum when whole genome sequence data was used for reconstruction of phylogeny [46]. However, phylogenetic analyses using whole genome data require curation with respect to recombination and insertion sites [47]. Also the use of whole genome phylogeny has been restricted due its compute-intensive nature. The phylogeny module of VirGen includes the whole genome phylogenetic trees obtained using a parallel version of the PHYLIP (Felsenstein, J., Department of Genetics, University of Washington, Seattle and Silicon Graphics Incorporation) implemented on SGI Onyx 300, a 4 processor parallel machine. The parameters for phylogenetic analysis in VirGen are as described previously [19]. Whole genome phylogenetic analysis facilitates identification of unclassified viruses and when coupled with the antigenicity data, assists in the identification of viral strains, isolated during epidemics.

**Figure 7:**
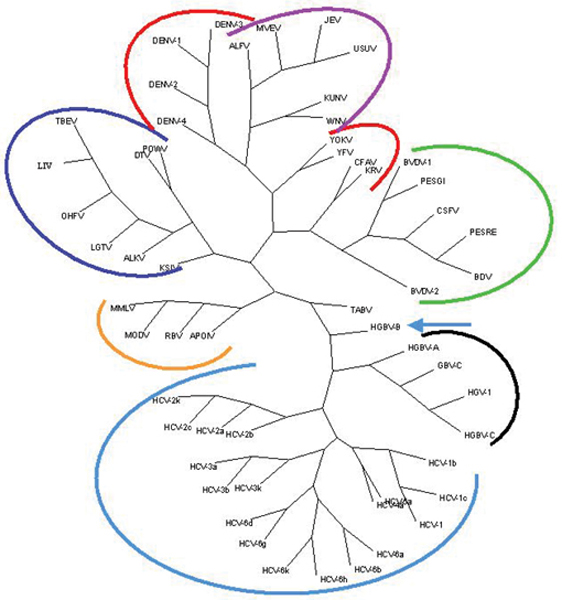
**Whole genome phylogenetic tree (unrooted) of family Flaviviridae reconstructed using maximum parsimony**. Colour coding for arcs is as follows. Red (Aedes borne Flaviviruses), Purple (Culex borne Flaviviruses), Blue (Tick borne Flaviviruses), Orange (No known vector Flaviviruses), Green (Pestiviruses), Cyan (Hepaciviruses) and Black (unassigned members of family Flaviviridae).

Reconstruction of a phylogenetic tree of family *Flaviviridae *generated using whole genome data is shown in the Figure 7. Confidence of the tree was evaluated using bootstrap data of 1000 replicates. The unrooted, most parsimonious tree obtained for 56 species belonging to the genera *Flavivirus, Pestivirus *and *Hepacivirus *clearly depicts the clustering of viruses as per their assigned genus. The tree also shows grouping of Flaviviruses into the mosquito-borne, tick-borne and no known vector clades. The mosquito-borne viruses show further separation into two clades namely, those which are transmitted through *Aedes *and through *Culex*. As can be seen from the figure 7, viruses transmitted by *Aedes *form two paraphyletic clades; one clade is made up of *Yellow fever virus *and *Yokose virus *and the other clade contains  *Dengue virus type I, II, III and IV*. *Cell fusing Agent virus *(CFAV) and *Kamiti River virus *(KRV), though transmitted by *Aedes *form a separate clade. Even though they share common ancestor with other mosquito-borne viruses, the fact that they are known to be insect-only and fail to replicate in the vertebrate cells or mice explains the observed branching pattern [48]. Viruses transmitted by *Culex *mosquito form a monophyletic clade. The branching pattern of the tick-borne Flaviviruses is gradual as compared to the dispersed branching of mosquito-borne clades. This could be attributed to the duration of life cycle of their respective vectors and vectors association with host [47]. *Tamana Bat virus *(TABV) branches out separately from rest of the *Flavivirus *members possibly due to its sequence divergence. The tree also shows that the *Pestiviruses *form a polyphyletic clade with the mosquito-borne *Flaviviruses*. *Border Disease virus *(BDV), *Pestivirus Reindeer *(PERSE) and *Classical Swine Fever virus *(CSFV) form a group where as *Bovine viral diarrhea virus-1 *(BVDV-1) and *Pestivirus Giraffe *(PESGI) group separately. *Bovine viral diarrhea virus-2 *(BVDV-2) branched out prior to the branching of other Pestiviruses. The branching of all six genotypes of *Hepatitis C virus *is also clearly seen. The tree shows that the unassigned members of *Flaviviridae *family form a polyphyletic clade with the Hepaciviruses, the outermost branch of which is *GB virus B *(GBV-B). The latest report of ICTV documents inclusion of *GBV-B *as a member of genus *Hepacivirus*. Phylogenetic tree reconstructed using the polyprotein data showed similar branching topology.

#### Genome to Vaccinome: compilation of predicted epitopes

Availability of genomic sequences has paved way for *in silico *design of vaccines, a field popularly known as 'reverse vaccinology' [49]. One of the prerequisites for *in silico *vaccine design is the availability of a curated data set of antigenic proteins and a set of programs for prediction of epitopes. As a step towards reverse vaccinology, VirGen stores predicted B-cell epitopes of antigenic proteins using sequence-based and structure-based algorithms developed in-house [50-52].

Kolaskar & Tongaonkar's method is a sequence-based approach for prediction of B-cell epitopes. The algorithm is based on antigenic propensity, which is assigned to each of the twenty amino acids depending on their frequency of occurrence in experimentally determined B cell epitopes. Parameters like hydrophilicity, accessibility and flexibility are averaged for every overlapping heptapeptide from N to C terminii and assigned to the central residue of every segment. The residues having average antigenic propensity ≥ 1.0 are termed as potential antigenic determinants. The accuracy of this method is ~75% and has been implemented in major sequence analysis packages viz, GCG [53] and EMBOSS [54]. VirGen contains the predicted epitopes of known antigenic proteins. However, if one wants to predict the B-cell epitopes for any other proteins of interest, using Kolaskar & Tongaonkar approach, a link is provided to the Antigenic program of EMBOSS.

**Figure 8:**
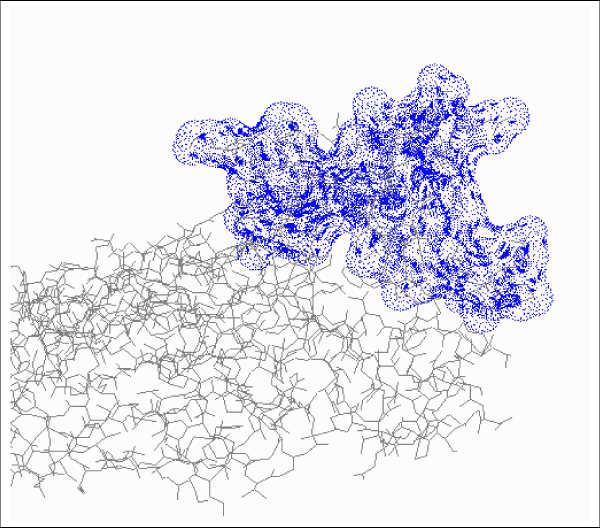
**Solvent accessible surface of predicted conformational epitope of envelope glycoprotein of Dengue 2 virus (PDB: 1OAN)**. The predicted conformational epitope contains two sequential epitopes and an individual accessible residue.

Conformational Epitope Prediction (CEP) algorithm developed in-house implements structure-based approach for prediction of sequential and conformational epitopes [37,51]. CEP server, for the first time allows mapping of antibody-binding sites on protein antigens with known structures [52]. The algorithm uses accessibility of residues and spatial distance cut-off. The major steps involve identification of at least three consecutive accessible residues to delineate antigenic determinants, extension of the same towards N and C terminii and prediction of conformational epitopes by collapsing antigenic determinants that are within the spatial proximity of 6Å. Accuracy of the algorithm is 75% when calculated using co-crystal structure data of antigen-antibody complexes from PDB. The CEP server provides a graphical interface for visualisation of predicted epitopes. Figure 8 shows solvent accessible surface of one of the predicted conformational epitopes of envelope glycoprotein of *Dengue virus II *(PDBID:1OAN) [55]. This conformational epitope is made up of two sequential epitopes 342–350 and 382–394. The antigenicity of the predicted epitope 382–394 has been experimentally validated previously [56]. The epitope predictions using the CEP algorithm are limited by the availability of three-dimensional structures of viral proteins. However, we strongly believe that with the structural genomics initiatives and availability of reliable approaches such as homology modeling and fold recognition, the three-dimensional structure data on viral proteins will no longer be a rate-limiting factor.

#### Module for Viral structures

Three-dimensional structural data is essential to understand function of proteins at molecular level. The 3D structures of proteins are known to be conserved and can accommodate sequence variation up to 80%. Structures of ~1082 viral proteins and viral assemblies have been solved and 3D coordinates are available in the PDB [57]. Structural genomics initiatives for viruses have been launched recently and are limited to a few viral species such as *SARS *[58] and *Poxvirus *[59]. VirGen includes a module for viral structures that are deposited in PDB. The PDB entries have been curated with respect to the description of organism source, as variations were found in the same. The viral structures can be searched using PDB ID as well as protein and organism description. Precomputed results of conformational epitope prediction or probable antibody-binding sites are also made available for these structures. The structure module facilitates identification of templates for knowledge-based homology modeling and identification of targets for structural genomics initiatives. Using homology-modeling approach, the structures of envelope glycoprotein of two strains of *Japanese encephalitis virus *have been predicted. These models were used to design candidates for peptide vaccine for Japanese encephalitis and helped to gain an insight into strain-specific variations [37,38].

### Comparative genomics to understand species-specificity

Analyses of curated data of whole genomes and proteomes reveals molecular variations, which when correlated with the observed phenotype provide a rationale for species-specific properties. As an attempt towards this, molecular mechanism of polyprotein cleavage at NS3-NS2B in JEV was modeled using the experimental data available for related flaviviruses since NS3 is a candidate for anti-viral therapy.

**Figure 9:**
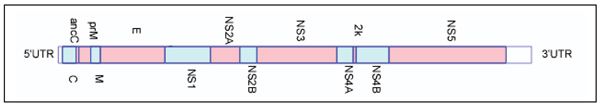
Genome organisation of Japanese encephalitis virus.

*Japanese Encephalitis Virus *(JEV), a member of *Flaviviridae *is a major causative agent of encephalitis in South-east Asia [60], the 11 Kb genome of JEV (Figure 9) codes for a polyprotein that is further, processed into 3 structural and 7 non-structural proteins by proteases of both, the host and the virus itself. NS3 has two domains, 178 residues at N' contains serine protease activity and ~450 residues at C' has helicase activity. The dual activity of NS3 increases its potential as a target for antiviral therapy. This viral protease is known to be highly efficient when associated with a hydrophobic stretch of NS2B [61,62]. The two-component protease is required for the *cis *cleavage of NS2A/NS2B and NS2B/NS3 as well as trans cleavage of NS3/NS4A and NS4B/NS5 [63,64]. Molecular mechanism of cleavage by NS3 has been studied in detail in Flaviviruses such as *Dengue type II *[65-67], *Hepatitis C virus *[68], *West Nile virus *[69] and *Alkhurma virus *[70]. Similar studies of NS3 in JEV are restricted to biochemical characterisation [71-73]. A detailed understanding of the molecular mechanism of cleavage by NS3 requires determination or simulation of the ternary complex comprising of NS3-NS2B-substrate. In the absence of crystal structure of JEV NS3, a comparative modeling approach was used to predict its structure. Since the functional protease is a two-component system, the structure of its cofactor, NS2B was predicted using molecular dynamics and its interactions were studied using docking simulations. Ternary complex of NS3-NS2B-substrate was then modeled to understand the underlying mechanism.

### Prediction of 3D structure of NS3

The 3D structure of NS3 serine protease domain of *Japanese Encephalitis virus *(*Virulent SA-14-14-2 strain*) was predicted using knowledge-based homology modeling approach. Two models of NS3 serine protease of JEV were built using the crystal structures of NS3 of *Dengue virus II *(DEN) PDB_ID: 1BEF [74] and *Hepatitis C Virus *(HCV) PDB_ID: 1JXP [75]. These templates showed remarkable similarity in terms of fold (two, six β barrel domains that are separated by a linker region), secondary structure content and conformation of active site. The sequence identity between NS3 of JEV with that of DEN and HCV is 47.16% and 13.3% respectively. The active site residues His51, Asp75 and Ser135 are found to be conserved. Sequence and structural alignments were used to identify the structurally conserved regions (SCR) and loops.

**Figure 10:**
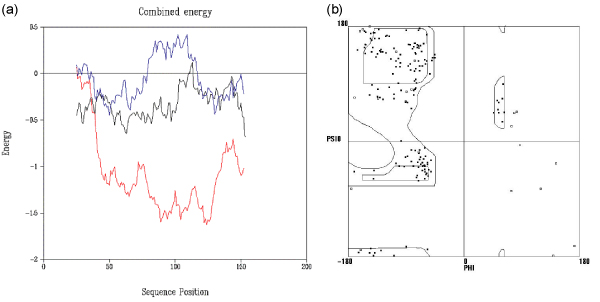
**Evaluation of predicted structure of NS3 of JEV**. (a) Plot of combined energy of 1BEF (blue), 1JXP (red) and model of NS3 (black) drawn using ProsaII (Sippl, 1993). (b) Ramachandran plot drawn using the MX program developed in-house (Kolaskar & Choudhuri, unpublished). Gly and non-Gly residues are shown using hollow and filled squares respectively.

The predicted structures were critically evaluated for stereochemical and geometrical correctness using multiple methods. Overall G factor score for both the models calculated using Procheck [76] was found to be in the range of -0.02 indicating that the models are well refined. Similarly, Z-scores calculated by ProsaII [77] were in the range typical for globular proteins. The plot of combined energy drawn using ProsaII indicated that the structure is energetically favourable (Figure 10a). Occupancy of (φ,ψ) angles in the Ramachandran plot [78] was found to be 100% for both the models. Furthermore, 91% occupancy was observed in the core regions of the Ramachandran plot, indicating the essential correctness of the predicted structures. The structural comparisons of both the models with their respective templates showed rmsd of 1.1Å and 1.8Å respectively. On the basis of evaluation of the models and the secondary structural content, the structure predicted using a template structure of *Dengue virus II *(1BEF) was accepted and used for subsequent studies (see Figure 10b).

### Modeling cofactor

A hydrophobic stretch of the non-structural protein 2B (NS2B), an integral membrane protein, serves as a cofactor for NS3 [61,72]. However, this sequence was not identified explicitly in JEV. A detailed analyses of the known cofactors of NS3 belonging to *Flaviviridae *family and their multiple sequence alignments combined with hydropathic profiles generated using Kyte & Dolittle index [79] helped to identify the putative hydrophobic stretch of NS2B. The residues, thus mapped for JEV are 62-WEMDAAITGSSR-73. It is known that the binding of the NS2B to NS3 is a co-translational event [65], where NS2B may only be partially folded. The structure of only hydrophobic stretch mentioned above was predicted using multiple MD simulations of 1ns duration. The peptide was found to adopt helical conformation predominantly (data not shown).

### Docking Cofactor & NS3

In the initial phase, docking of NS3 serine protease with the cofactor was carried out to study their interactions at molecular level. The cofactor (NS2B) was first docked onto NS3, followed by docking of substrate with the complex of NS2B-NS3.

**Figure 11:**
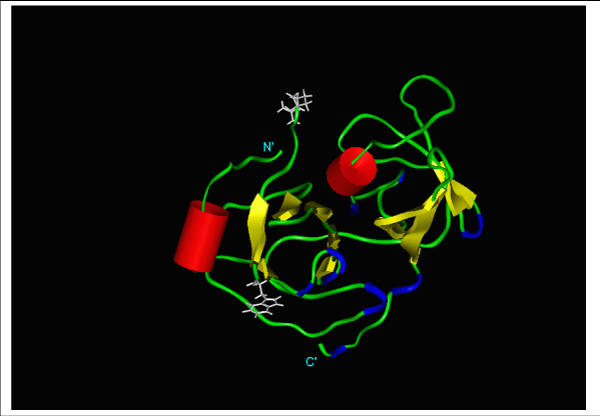
**Kabsch & Sander secondary rendering of the predicted structure of NS3-NS2B complex**. N' and C' terminii of NS3 are shown. The atoms of N' Trp and C' Arg of the cofactor, NS2B are shown using white stick rendering. Note: The strand of NS2B forms one of the blades of the β propeller domain of NS3.

The Kabsch & Sander secondary rendering of predicted structure of NS3 and NS2B complex is shown in the Figure 11[80]. As can be seen, the binding of NS2B helps in stabilising the structure of NS3 by formation of intermolecular hydrogen bonds. Out of 12 amino acid residues of NS2B, 6 were found to form 8 the hydrogen bonds with 6 residues of NS3. The NS2B peptide in the complex was found to adopt extended conformation in place of a helix. The extended conformation of NS2B contributes to the formation β- propeller-like domain in NS3 by serving as one of the blades of the propeller.

### Simulation of ternary complex

The exquisite selectivity of serine proteases for particular substrate is a result of the existence of specific binding sites on the enzyme for amino acid side chains of the substrate [81]. The substrate is oriented by binding of the amino acid side chain of the P1 residue in the S1 pocket (P1 is the substrate residue at the amino terminal, and P1' is the residue at the carboxyl terminal of the scissile bond) via hydrogen bond. It is known that the substrate binding cleft of the Dengue virus II protease is not very extensive and does not appear capable of providing specific interactions in the absence of NS2B activating peptide, with side chains beyond P2 and P2' [74]. This observation is consistent with the heterogeneity of residues beyond these sites as seen in flaviviral proteases.

**Figure 12:**
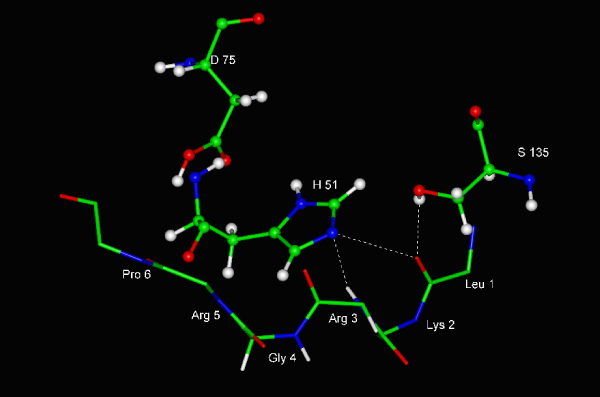
**Interactions of NS3 with its substrate at the binding site**. Active site residues of NS3 are shown in ball & stick rendering. The backbone of the substrate is shown in stick rendering. The dotted lines indicate hydrogen bonds between substrate and NS3. Residue colouring is as per atom types.

NS3 of JEV cleaves the polyprotein at specific locations having the sequence KR-GR (site between NS4B and NS5) apart from other known cleavage sites. In the absence of structural information of the substrate, the structure of six residues (LKRGRP) flanking on either side of the NS4B/NS5 cleavage site was predicted using the MD strategy described for NS2B. The ternary complex was obtained by docking of substrate to NS3-NS2B complex using the docking protocol mentioned above. Of the 100 docked structures, 23 were selected based on geometry and the interactions with NS3. It was observed that the van der Waal interactions contributed maximally towards the overall interaction energy. The active site residues His51 and Ser135 were found to interact with Leucine of the substrate via the formation of hydrogen bonds (see Figure 12). The two Arginine residues of the substrate also interact with Thr52 and Thr54 of NS3, both of which are part of the binding site.

**Figure 13:**
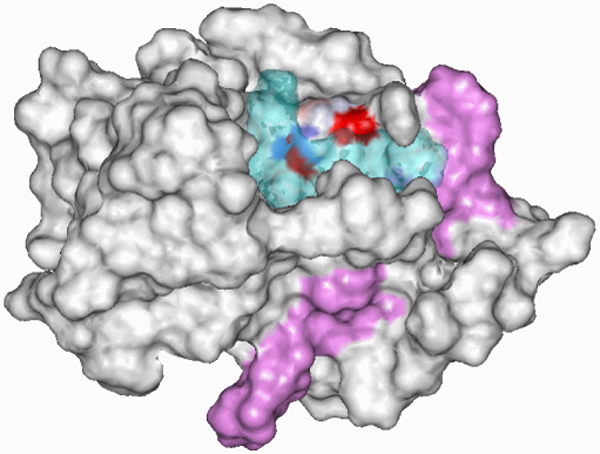
**Connolly surface rendering of ternary complex (NS3-NS2B-substrate)**. The surface of NS3 is coloured according to charge spectrum. The surface of cofactor is shown in pink. The surface of substrate is shown in cyan with reduced transparency to facilitate viewing of the charges in the active site

Table 3 shows the amino acid residues of NS3 binding pocket of DEN and JEV respectively. As can be seen in the Figure 13, the binding pocket of NS3 of JEV has a deep curvature as compared to DEN-NS3. The entire surface is of NS3 except for the binding pocket is neutral. The localization of charged residues in the binding pocket is shown in Figure 13. The substrate i.e., side chain atoms of Pn residues of the polypeptide interact with the binding pocket residues i.e., Sn thereby providing conducive environment for cleavage. The variation in the binding site residues of *Dengue virus II *NS3 and JEV NS3 (see Table 3) may account for the species-specific variation of the microenvironment. Thus, even though the overall fold of the protein is similar to other serine proteases, the microenvironment of the binding pocket accounts for its specificity. Such variations have implications in the design of antiviral drugs.

## Conclusion

The reductionalist approach such as whole genome sequencing enables understanding of life and its processes at molecular level. However, the challenge in the post-genomic era is to establish the link between the genomic parts and phenotypic features, which requires systematic organisation and integration of various databases that span the spectrum of Biocomplexity. VirGen is a comprehensive genomics resource for viruses. VirGen attempts to compile and curate the whole genome sequences of viruses. Various features and utilities to analyse viral genome data has been discussed using a case study of family *Flaviviridae*. The NS3 case study involving prediction of structure of enzyme, cofactor and docking of cofactor and substrate to explain molecular mechanism of cleavage in JEV demonstrates the utility of resources such as VirGen in studying the species-specific variations. Comparative genomics studies of this kind enable expansion of the available knowledgebase. The primary as well as the value-added derived data in VirGen will be highly useful not only in understanding the strain and species-specific variations but will also serve as starting point for discovery of diagnostic kits, antiviral drugs and vaccines. Furthermore, we believe that it would be a useful resource in analysing the outcome of metagenomics initiatives [82,5].

## Methods

### Data curation & annotations

Various sequence-based [27,31,32,50] and structure-based [37,51,52] bioinformatics approaches were used to curate, annotate and analyse viral genome sequence data. Perl scripts were used to retrieve, parse, populate and update the database.

### Homology modelling: NS3

The models were built using Homology module of Insight II molecular modeling package. Amber-all atom force field [83] and distance dependent dielectric constant of 4rij was used. The models were refined using steepest descents and conjugate gradient methods, the detailed protocol of which is described previously [37,38].

### Molecular dynamics simulations: NS2B peptide (co-factor)

The initial conformation of the peptide WEMDAAITGSSR was assigned randomly from the allowed region of the Ramachandran plot. The equilibration was carried out for 100ps. Amber force field v1.6 and a distance dependent dielectric constant of 4rij was used. The trajectory data was sampled every 10ps, resulting in 100 frames per MD run. The conformation that is most populated, having the least energy and lying in the allowed region of Ramachandran plot was identified for docking studies.

### Molecular docking simulations: secondary and ternary complexes

Docking studies were carried out using the Affinity module of InsightII.

The protocol used is as follows:

▪ Amber force field with implicit solvent model was employed.

▪ Initially the ligand (NS2B/substrate) was placed near the binding site.

▪ The residues of protein lying within 5Å radius from the center of ligand were defined as 'movable atoms' whereas the rest of the molecule was kept rigid during docking and was defined as 'bulk set'.

▪ Ligand was flexible with respect to (φ,ψ) torsional angles. Hydrogen bond donor and acceptor atoms were defined for both the protein and the ligand. In order to avoid displacement of the ligand far away from the active site residues, the ligand was confined to 3Å radius.

▪ Initial coarse search was carried out using Quartic_vdw_no_Coulb method in which coulombic terms are not calculated thereby excluding electrostatic interactions. This allows sampling of larger conformational space in a shorter time interval. 100 docked conformations were collected during this phase in which the ligand is subjected to random combinations of translational and rotational movements followed by 1000 steps of minimization using conjugate gradients. The conformations lying within the energy range of 100 kcal and 1Å RMS deviation were selected.

▪ The conformations collected in previous step were filtered using various parameters like orientation of ligand, distance from the active-site residues and bad contacts, if any.

▪ Twenty conformations satisfying above criteria were further refined in the second stage using Group_based method in which non-bond interactions were calculated using van der Waals and coulombic cutoffs of 15 and 10Å respectively. The conformers so obtained were refined by minimization of 100 steps of conjugate gradient followed by 50 steps of simulated annealing starting at an initial temperature of 500 K up to a final temperature of 300 K with temperature leap of 4 K at each step. The Newton's equations of motion were integrated using the Verlet algorithm [84] with a time step of 1fs using NVT ensemble. Temperature control was achieved by direct scaling of atom velocities. Finally, the conformers were minimized to a gradient of 0.001 kcal/mole/Å or less using conjugate gradients.

▪ The conformers were evaluated based on the interaction energy, which is a sum total of van der Waal and coulombic interactions. The least energy conformer having favorable interactions with the residues in the binding pocket was selected for further analysis.

## Authors' contributions

UKK: Design and coordination of the study, analyses and interpretation of data, drafted the manuscript

SGB: Development of database & query interface, docking studies

GSM: Whole genome phylogeny, docking studies and drafted manuscript

MJ: Homology modeling of JEV NS3

SB: MSA and derivation of signature sequences

ASK: Conceived the study, critical evaluations and useful suggestions

## Figures and Tables

**Table 1:** Growth statistics of VirGen.

Sr. No	Release	No. of families	No. of genomes
1	1.0	8	559
2	1.1	8	846
3	1.2	10	905
4	1.3	10	987
5	1.4	15	1158
6	1.5	20	1444
7	1.6	25	2290
8	1.7	25	2475
9	1.8	25	2616
10	1.9	25	2791
11	2.0	25	2895

**Table 2:** Families in VirGen database listed according to type of genetic material and host range.

Family	Genetic material type	Host
Arteriviridae	ssRNA(+)	Vertebrates
Astroviridae	ssRNA(+)	Vertebrates
Barnaviridae	ssRNA(+)	Fungi
Caliciviridae	ssRNA(+)	Vertebrates
Coronaviridae	ssRNA(+)	Vertebrates
Dicistroviridae	ssRNA(+)	Invertebrates
Flaviviridae	ssRNA(+)	Vertebrates
Leviviridae	ssRNA(+)	Bacteria
Luteoviridae	ssRNA(+)	Plant
Picornaviridae	ssRNA(+)	Vertebrates
Togaviridae	ssRNA(+)	Vertebrates
Tombusviridae	ssRNA(+)	Plant
Tymoviridae	ssRNA(+)	Plant
Narnaviridae	ssRNA(+) Naked	Fungi
Bornaviridae	ssRNA(-)	Vertebrates
Filoviridae	ssRNA(-)	Vertebrates
Paramyxoviridae	ssRNA(-)	Vertebrates
Rhabdoviridae	ssRNA(-)	Plant, Vertebrates
Hypoviridae	dsRNA	Fungi
Totiviridae	dsRNA	Protozoa
Circoviridae	ssDNA	Vertebrates
Microviridae	ssDNA	Bacteria
Papillomaviridae	dsDNA	Vertebrates
Polyomaviridae	dsDNA	Vertebrates
Tectiviridae	dsDNA	Bacteria

**Table 3:** Residues in the binding pocket of NS3 of JEV and DEN-2. Active site residues are underlined and variations are shown in bold.

Binding pocket	JEV	DEN2
S2	H51	H51
	D75	D75
	G151	G151
	N152	N152
	G153	G153
S1	**L115**	**V115**
	S131	P131
	S135	S135
	G136	G136
	Y150	Y150
	S163	S163
S1'	Q35	Q35
	**I36**	**A36**
	H51	H51
	V52	T52
	S135	S135
S2'	Q35	Q35
	**I36**	**A36**
	**P132**	**R132**
	G133	G133
Hydrophobic cluster	**L115**	**V115**
	F116	F116
	V126	V126
	**A160**	**S160**
	Y161	Y161
